# Marine Biotoxins in Whole and Processed Scallops from the Argentine Sea

**DOI:** 10.3390/md20100634

**Published:** 2022-10-10

**Authors:** Alejandra B. Goya, Danial Baqer, Ryan P. Alexander, Patrycja Stubbs, Karl Dean, Adam M. Lewis, Lewis Coates, Benjamin H. Maskrey, Andrew D. Turner

**Affiliations:** 1 Marine Biotoxin Department, Mar del Plata Regional Laboratory, National Service for Agri-food Health and Quality (Senasa), AvisoDorrego y Víctimas del ‘46, Puerto, Mar del Plata B7600, Buenos Aires Province, Argentina; 2 Centre for Environment, Fisheries and Aquaculture Science (Cefas), Barrack Road, The Nothe, Weymouth, Dorset DT4 8UB, UK; 3 Department of Biological Sciences, University of Surrey, Stag Hill, Guildford GU2 7XH, UK

**Keywords:** paralytic shellfish poisoning, lipophilic toxins, domoic acid, liquid chromatography, LC-MS/MS, toxicity and toxin profiles

## Abstract

Harmful algal blooms are an increasing worldwide threat to the seafood industry and human health as a consequence of the natural production of biotoxins that can accumulate in shellfish. In the Argentine Sea, this has been identified as an issue for the offshore fisheries of Patagonian scallops (*Zygochlamys patagonica*), leading to potentially harmful effects on consumers. Here we assess spatial and temporal patterns in marine biotoxin concentrations in Patagonian scallops harvested in Argentinian waters between 2012–2017, based on analyses for paralytic shellfish toxins, lipophilic toxins, and amnesic shellfish toxins. There was no evidence for concentrations of lipophilic or amnesic toxins above regulatory acceptance thresholds, with trace concentrations of pectenotoxin 2, azaspiracid 2 and okadaic acid group toxins confirmed. Conversely, paralytic shellfish toxins were quantified in some scallops. Gonyautoxins 1 and 2 dominated the unusual toxin profiles (91%) in terms of saxitoxin equivalents with maximum concentrations reaching 3985 µg STX eq/kg and with changes in profiles linked in part to seasonal changes. Total toxin concentrations were compared between samples of the adductor muscle and whole tissue, with results showing the absence of toxins in the adductor muscle confirming toxin accumulation in the digestive tracts of the scallops and the absence of a human health threat following the processing of scallop adductor meat. These findings highlight that paralytic shellfish toxins with an unusual toxin profile can occur in relatively high concentrations in whole Patagonian scallops in specific regions and during particular time periods, also showing that the processing of scallops on board factory ships to obtain frozen adductor muscle is an effective management process that minimizes the risk of poisonings from final products destined for human consumption.

## 1. Introduction

In the aquatic ecosystem, photosynthesising organisms such as phytoplankton form the basis of marine food webs. Overgrowth of these organisms results in algal blooms; natural phenomena that occur as part of the seasonal cycle in the marine ecosystem [1,2]. Some phytoplankton species known as harmful algal blooms (HABs) can naturally produce biotoxins which can be harmful to human, animal and environmental health. These biotoxins are wide-ranging in terms of chemical structure, physicochemical properties, mechanisms of toxicity and toxicological impact. Multiple exposure routes exist, with the most commonly occurring human health threat resulting from the consumption of seafood such as bivalve shellfish [3]. Bivalves, including scallops such as *Z. patagonica*, are filter feeders and thus commonly accumulate toxins from phytoplankton in their viscera such as the digestive tract and are poorly bound to other tissues such as the adductor muscle [4]. In general, biotoxins will have limited effects on the health of shellfish but severe human intoxication is possible upon consumption, potentially resulting in human intoxication syndromes including paralytic shellfish poisoning (PSP), Diarrheic shellfish poisoning (DSP) and Amnesic shellfish poisoning (ASP). Shellfish contaminated with marine biotoxins not only pose a risk to human health but also can have a drastic effect on the local, regional, and national economy via closures of harvesting sites and disruption to food supplies and logistics [5]. Routine monitoring of these toxins is therefore essential and in most parts of the world a regulatory requirement.

**Figure 1:**
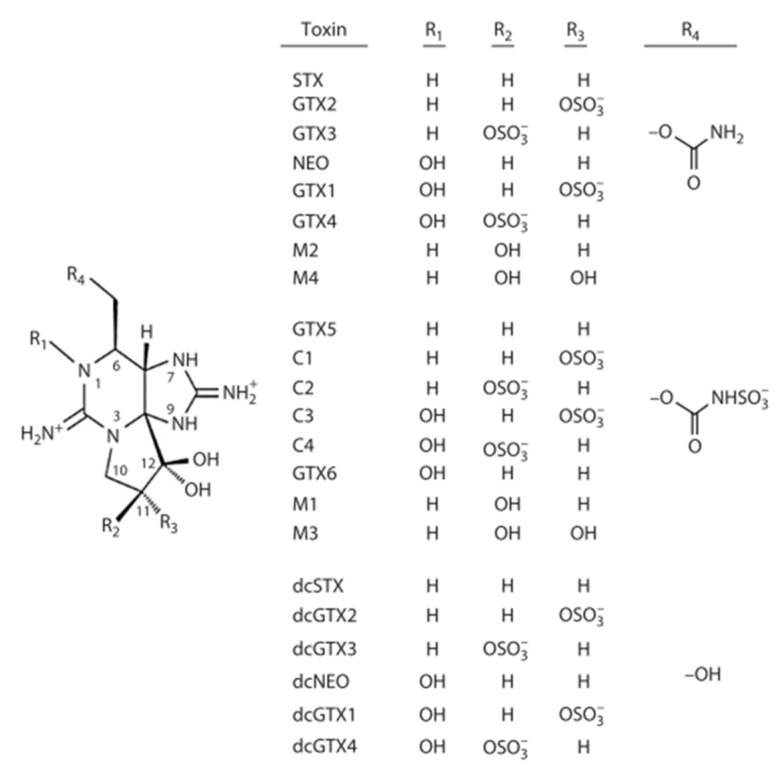
Structures of paralytic shellfish toxins; saxitoxin and the analogues incorporated into the analysis method, based on [10].

Paralytic shellfish toxins (PSTs) are a family of toxins related to saxitoxin (STX) primarily produced by dinoflagellates of the genus *Alexandrium*, *Gymnodinium* and *Pyrodinium* [6,7,8,9]. Saxitoxins (STXs), a family of >56 analogues, are hydrophilic toxins which are classified based on the three common functional groups at R4 (Figure 1), carbamoyl (e.g., STX, GTX2), N-sulfocarbamoyl (e.g., GTX5, C toxins) or carbamoyl (e.g., dcSTX, dcGTX2) [10]. Different substituents result in variable toxicities, with the carbamoyl congeners typically exhibiting the highest toxicity, and the N-sulfocarbamoyl toxins the lowest. PSTs block the voltage-gated sodium channels in nerve cells by utilizing the positively charged guanidinium group to bind to the carboxyl group at site 1 of the sodium channel [11]. This disrupts the communication between nerve cells resulting in cramps, numbness, and paralysis of rudimental body functions such as respiration; potentially causing death given high enough exposure [9,12,13]. PSTs, along with other regulated marine toxins, must be monitored in live bivalve molluscs in order to determine whether shellfish harvesting areas can open for the sale of shellfishery products. To this end, maximum permitted limits (MPLs) exist, which for PSTs equates to a safety threshold of 800 µg STX eq/kg [14]. PSTs can be monitored in shellfish using a range of detection methods [15] such as the mouse bioassay (MBA), receptor binding assay (RBA), liquid chromatography with fluorescence detection (LC-FLD) methods [16,17] and LC with tandem mass spectrometry (LC-MS/MS) [18], with the pre-column oxidation LC-FLD method based on AOAC method 2005.06 the current reference method in the European Union for official control testing [19,20,21].

In relation to other toxin-producing phytoplankton, the diatom *Pseudo-nitzschia* spp., is the source of Domoic acid (DA), which when accumulated in shellfish tissue to high enough concentrations can result in ASP. DA and one of its isomers, epidomoic acid, are hydrophilic cyclic amino acids which behave as a glutamate agonist, interfering with neurotransmissions within the brain and leading to gastrointestinal symptoms, confusion, short-term memory loss, coma and breathing difficulties [22]. Detection of DA in shellfish is straightforward as the conjugated dienes allow its detection with a UV detector [23] coupled with liquid chromatography (LC-UV), or by LC-MS/MS [24]. The EU MPL for official control testing is defined as 20 mg/kg [20].

The third and final group of EU-regulated toxins, the ‘lipophilic toxins’ (LT) are the fat-soluble marine biotoxins which have been linked to toxicity in both humans and mice. DSP is the most frequently occurring intoxication from marine shellfish toxins resulting from the consumption of shellfish contaminated with the okadaic acid group (OA-group) toxins: Okadaic acid (OA), Dinophysis toxin 1 and 2 (DTX1&2) and the ester derivatives, as well as the potential inclusion of the Pectenotoxin group (PTXs) [25,26]. OA-group toxins inhibit protein phosphatases in cells which upon ingesting contaminated shellfish can cause symptoms such as nausea, vomiting, diarrhoea and abdominal pain [27]. Other regulated LTs include Yessotoxins (YTX) and Azaspiracids (AZA) [28], with the latter responsible for Azaspiracid Shellfish Poisoning (AZP) [29]. Cyclic imines such as Gymnodimine (GYM), Spirolides (SPX) and pinnatoxins (PnTx) are not regulated but their lipophilic nature and high toxicity in mice [30] following intraperitoneal injection have resulted in an increasing interest in monitoring these compounds. The European Union reference method utilizing LC-MS/MS is used in most countries for routine testing of bivalves for the entire suite of LTs, with MPLs of 160 µg/kg for OA-group toxins combined, AZAs together and 3.75 mg/kg for YTXs together [31].

The Argentine Sea is in the Southwest Atlantic Ocean off the southeastern coast of Argentina. Geographically, its limits extend from the mouth of the Río de La Plata (35° LS) in the north to Isla de los Estados in the south, and from the Argentine coastline (approximately 5000 km) to the 200-m isobath. The sea has an approximate extension of 1,000,000 km². The sea is a highly productive marine zone, resulting in an active seafood fishing industry. Mussels (*Mytilus edulis* and *Aulacomya ater*), scallops (*Aequipecten tehuelchus* and *Z. patagonica*), and oysters (*Crassostrea gigas* and *Ostrea puelchana*) are the commonly harvested bivalve species, as well as various clam (*Ameghinomya antiqua* and *Panopea abbreviata*), razor (*Ensis macha* and *Solen tehuelchus*) and gastropod species (*Zidona dufresnei* and *Adelomelon beckii*) [32,33]. *Brachidontes rodriguezii* is a smaller coastal mussel which is often used as a sentinel species [34] in regulatory monitoring programs within the country, as it is dominant in the intertidal along the Argentinian coastline. Whilst the majority of bivalve species are harvested from in-shore coastal waters along the Argentinean coastline, such as the scallop species *A. tehuelchus* which inhabits shallow shelf bottoms along the Argentinian coastline [35], scallops such as *Z. patagonica* are typically fished from off-shore regions, sometimes miles into the ocean and therefore may not follow typical trends of toxification shown by sentinel species and those harvested on the coast.

In the Argentine sea, algal-produced marine biotoxins including PST, LT and ASP have been reported. The first LT outbreak in Argentina occurred in 1999 from the sale of mussels harvested from the San José Gulf and Nuevo Gulf [32] with numerous outbreaks since then containing a range of toxins; OA, DTX-1, DTX-2, PTX-2, YTX, and AZA [33,36]. YTX was detected in harvested scallops from Buenos Aires and Santa Cruz and AZA-2 in mussels (*Brachidontes rodriguezii*) and yellow clams (*Mesodesma mactroides*) [37]. SPX and GYM are not included in the list of regulated toxins in Argentina [33], however, SPX has been detected in very low concentrations along the entire coast of Argentina [37,38] and no GYM has been detected in the Southwest Atlantic [33]. Despite no ASP outbreaks in Argentina since its discovery, DA detection is apparent from north to south in both coastal and oceanic areas [33]. Ref. [39] reported DA detection in phytoplankton sampling in the San Matías Gulf and Nuevo Gulf. DA has also been detected recently in the Southern Right Whales (*Eubalaena australis*) in the Península Valdés, Golfo Nuevo and Golfo San José [40] evidencing a threat to animal health from DA in Argentine waters.

PST-producing algal blooms have been found along the coast of Argentina since 1980 [41,42], with dense blooms of *Alexandrium catenella* found in the colder waters of the south (Patagonian regions such as Chubut and Beagle Channel) and *Gymnodinium catenatum* in more northern warmer waters (province of Buenos Aires) [42,43,44,45]. Several PSP outbreaks have been documented since the 1980s in Argentina (summarized by [32]). In Argentina, six species are associated with the production of OA, DTX and PTX: *Dinophysis acuminata*, *D. caudata*, *D. tripos*, *D. fortii*, *D. norvegica* & *Prorocentrum lima* [33]. YTX producers in Argentine waters are as follows: *Lingulodinium polyedra, Gonyaulax spinifera*, and *Protoceratium reticulatum* [46]. Blooms of AZA-producing species have been documented in Argentina and include *Azadinium spinosum*, *A. poporum, A. languida, A. dexteroporum* & *A. luciferelloides* [33,47,48]. An SPX-producing species *Alexandrium ostenfeldii* has also been found. Nine species of toxic *Pseudo-nitzschia* (*seriata, australis, multiseries, pungens, delicatissima, fraudulenta, pseudodelicatissima, turgidula,* and *brasiliana*) have been reported in the Argentine sea [33].

The National Service for Agri-food Health and Quality (SENASA) is the authority responsible for establishing the sanitary regulations that apply to the monitoring of bivalve molluscs destined for human consumption. Monitoring of toxin levels in bivalves is conducted using the MBA for PSP and LC-UV is used for ASP tests [49]. For LT, the LC/MS-MS method is used for shellfish destined for export, and the MBA is applied for shellfish monitoring destined for internal market or sanitary surveillance on coasts. Instrumental chemical testing methods have now become more widely available for global regulatory testing of LT and PSP such as chromatographic separation with fluorescence detection [16,17,50,51] or more advanced chromatographic methods utilising highly-sensitive mass spectrometers [31,49]. Such analytical methods provide both an accurate determination of toxin concentrations as well as additional data on toxin profiles. These data enable insights to be generated regarding the level and variability of toxicity and toxin profiles throughout geographical regions and over time [49,52], significantly improving the understanding of toxin presence and aiding risk assessment programmes.

**Figure 2:**
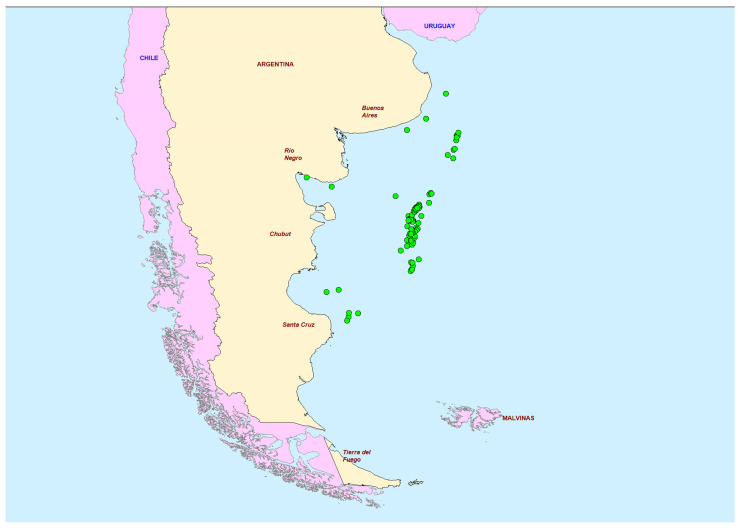
Sampling locations for scallop samples from this study in the Argentine sea.

This study aimed to analyse scallop samples for the presence of PSTs, LTs and domoic acid.. Upon receipt at Cefas, samples were assigned unique sample IDs with no prior analysis of scallop samples for the presence of PSTs, LTs, and domoic acid. Initially, a small number of samples were analysed including the two previously commercially-important Argentine scallop species, *Z. patagonica*, and *A. tehuelchus*. Subsequently, a large number of *Z. patagonica* samples were collected from various offshore sites in the Argentine sea (Figure 2) between 2012–2017 for the main study. No *A. tehuelchus* samples could be incorporated into the main study, unfortunately, as by this time, the species was no longer collected commercially from in-shore coastal waters of the Buenos Aires Province. A total of 189 samples were analysed, comprising 96 whole scallops and 93 samples containing adductor muscle knowledge of origin or harvesting date. Total PST and LT content was determined using LC-MS/MS and total DA content was determined by LC-UV. Results obtained from LC-MS/MS and LC-UV enabled the assessment of sample toxicity and associated toxin profiles and the potential link to geographic or temporal sources within Argentinian waters. The aim was to generate evidence for the levels and geographical distribution of toxicity potentially found in offshore scallops, the toxin profiles associated with contaminated organisms, and the effects of scallop processing on toxin loading.

## 2. Results

### 2.1. Paralytic Shellfish Toxins

#### 2.1.1. Initial Assessment

Prior to the main study, archived scallop samples were assessed to determine the likelihood of toxin presence in either whole tissue or adductor meat after processing. A total of seven scallop samples were initially analysed by LC-MS/MS to quantify PST concentrations and estimate total PST toxicity in µg STX eq/kg, comprising a total of twelve samples when five of these were received as “separate whole” and “adductor only” portions.

A total of four *A. tehuelchus* samples were tested, comprising three whole scallop sub-samples and four adductor sub-samples. As such, three of the samples were split into both adductor and whole scallop tissue, allowing a comparison of toxin concentrations between adductor and whole tissue (Table 1). The sample harvested on 3 October 1986 exhibited high toxicity (>14,000 µg STX eq/kg) in the whole tissue. Results from the adductor from the same sample still showed toxin concentrations above the MPL of 800 µg STX eq/kg (854 µg STX eq/kg). Similarly, the other two samples harvested on 28 October 1986 and 17 October 2000 also contained high PST with total concentrations of ~4000 µg STX eq/kg in whole animals, and 1289 and 101 µg STX eq/kg in the adductor, respectively. Results from the remaining *A. tehuelchus* sample (adductor only) contained only a trace level of PSTs. Consequently, out of the four samples received in both whole and adductor-only format, the proportion of toxins quantified in the adductor in comparison to whole tissues ranged from 1.0% to 26.5%.

Three *Z. patagonica* samples were analysed comprising three whole tissue samples and two adductor-only sub-samples. The three whole samples showed either low detectable toxins (27 January 2012) or total concentrations below the MPL (94.5 and 185 µg STX eq/kg for samples harvested on 20 October 2009 and 14 June 2011, respectively). Undetectable or very low concentrations of PSTs (<2.0 µg STX eq/kg) were quantified in the adductor sub-samples. The sample taken on 14 June 2011 which contained 185 µg STX eq/kg in the whole tissue was found to have just 1.8 µg STX eq/kg in the adductor sub-sample (Table 1).

#### 2.1.2. Main Study

**Figure 3:**
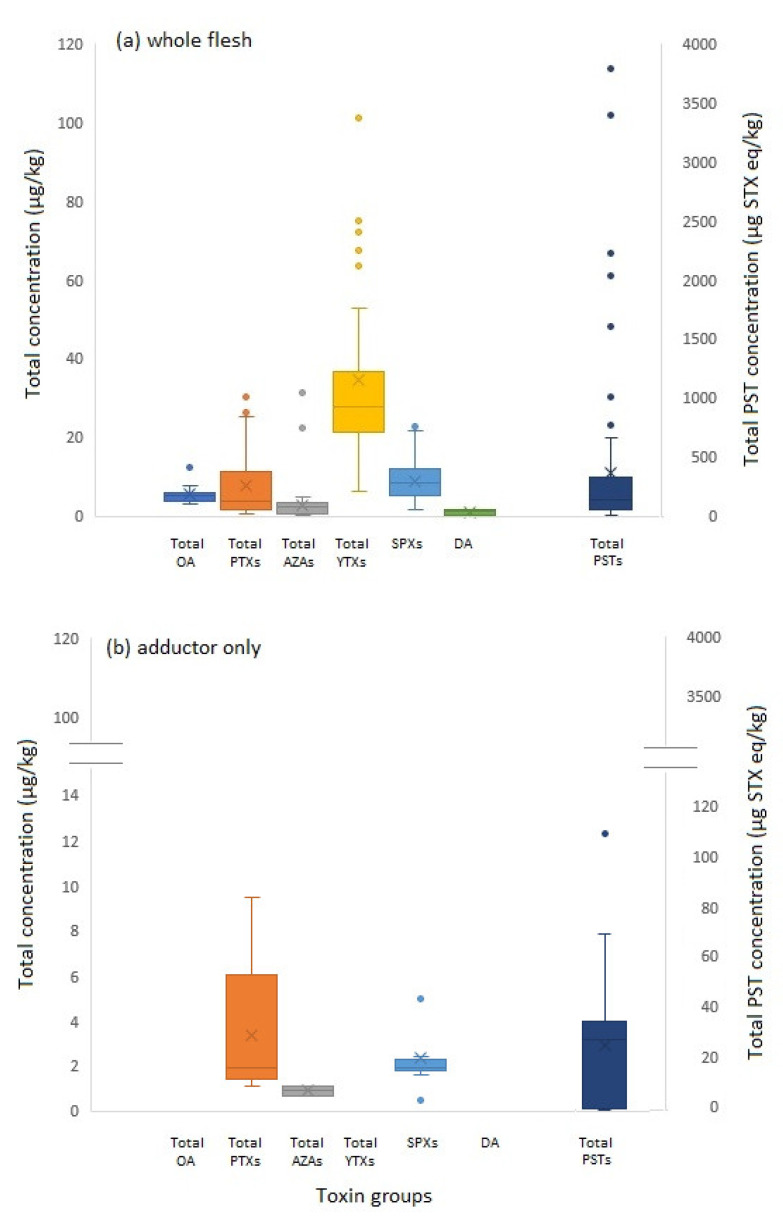
Box and whisker plots illustrating the range of total toxin group concentrations quantified in (**a**) whole scallop and (**b**) adductor tissues, showing OA-group, PTXs, AZAs, YTXs, SPXs and DA on primary vertical axis (µg/kg), and total PSTs (µg STX eq/kg) on secondary axis.

Of the 96 whole *Z. patagonica* scallop samples tested, 71 (74%) were found to contain PSTs at concentrations above 1 µg STX eq/kg, the mean limit of detection (LOD) for the validated method. Total toxin concentrations were highly variable ranging from 1 to >3000 µg STX eq/kg as shown by the box and whisker plot (Figure 3). Six of these samples exhibited total PST concentrations higher than the MPL, reaching a maximum of 3782 μg STX eq/kg. From the adductor muscle sub-samples only 14 (15%) had detectable levels of PSTs, ranging between 1 and 71 μg STX eq/kg. From these adductor samples, the mean ratio of total PST in adductor vs whole tissue was 22 ± 20%, with proportions ranging from 8% to a maximum of 73%. The table of actual concentrations across all samples is tabulated in Appendix A.

**Figure 4:**
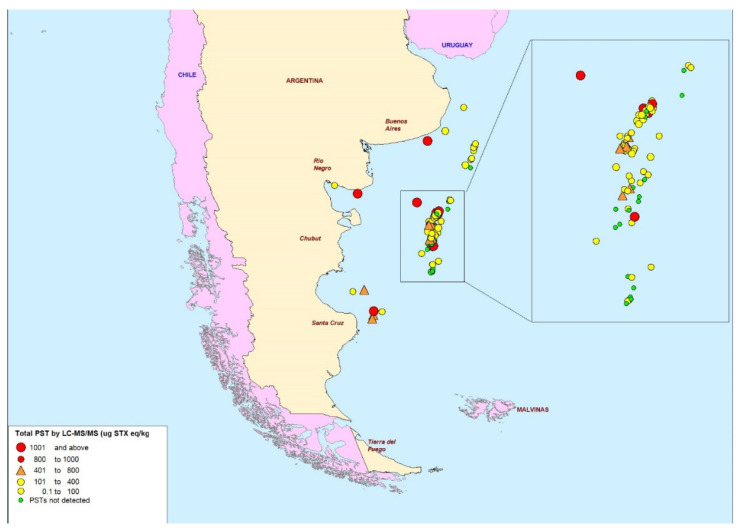
Map of scallop sampling points showing indications of total paralytic shellfish toxin levels as determined by LC-MS/MS (μg STX eq/kg).

Significant variation in total PSTs was quantified between both different and similar geographical regions within the country (Figure 4). In terms of geographical variability, a cluster of varying PST concentrations was observed offshore in the centre of the Argentinian sea, east of Chubut Province (zoomed-in region in Figure 3), primarily dominated by samples <400 µg STX eq/kg. Sampling locations located in the northern deep-water region of the Argentine sea, north of the central offshore region, all contained trace concentrations <400 µg STX eq/kg. Six out of the eleven samples analysed from close to Argentina’s coastline were >400 µg STX eq/kg and the remaining five still showed detectable levels of PST.

**Figure 5:**
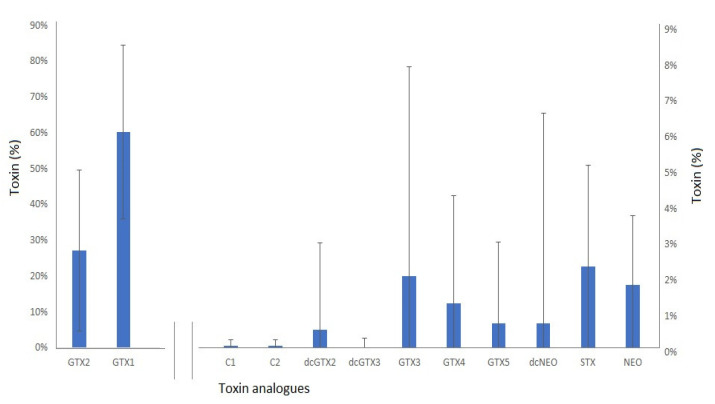
Mean paralytic shellfish toxin profile (percent of total STX eq) ± s.d. in *Z patagonica* scallops (*n* = 71), showing GTX1 and GTX2 on the primary y-axis, and remaining analogues on the secondary.

The mean toxin profile (Figure 5) determined across all *Z. patagonica* samples was dominated by gonyautoxins, with GTX1 on average making up to 63% of the profile in terms of saxitoxin equivalents, followed by GTX2 (28%). Smaller relative concentrations (1–3%) of dcGTX2, GTX3, GTX4, GTX5, STX and Neo were also quantified, together with trace amounts (<1%) of other analogues. The standard deviations (SD) associated with the mean toxin proportions were found to be large, however, indicating significant variability of the mean profile across the whole sample set.

**Figure 6:**
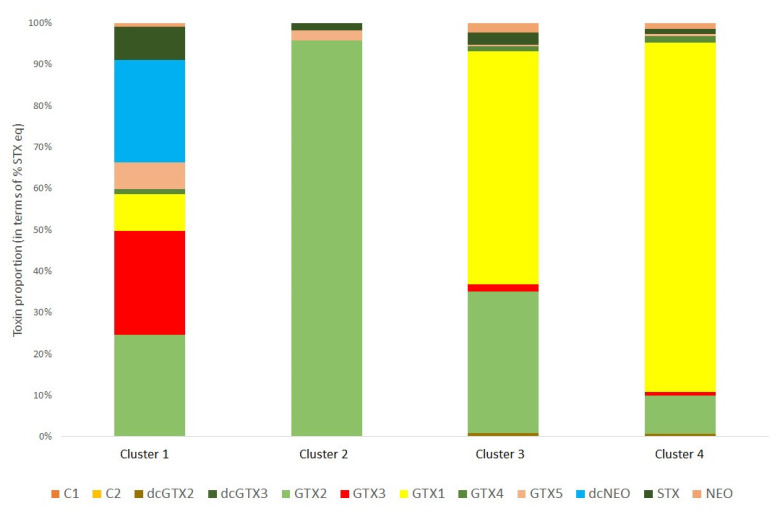
Mean toxin profile determined for each cluster (cluster 1, *n* = 2; cluster 2, *n* = 4; cluster 3, *n* = 38, cluster 4, *n* = 27) for the scallops harvested and analysed for paralytic shellfish toxins.

This wide variability in profiles across the samples infers that other factors may affect the profiles of individual samples. Therefore, a K-means cluster statistical analysis was conducted to determine clusters of samples with specific profile types, with each cluster showing similar toxin profiles. In total four clusters were identified as illustrated in Figure 6, with the majority of samples falling into either cluster 3 or cluster 4. Cluster 1 was a profile associated with just two samples (2.8%) containing a wide range of toxin analogues, specifically GTX1-5 and STX with trace amounts of NEO. Four scallop samples (5.6%) exhibited a cluster 2 profile, which all showed a near total dominance of GTX2 with just trace amounts of GTX5 and STX. Cluster 3 was associated with 38 scallops (54%) containing both GTX2 and GTX1 at high relative proportions and only trace relative concentrations of other GTXs, STX and NEO. Finally, 27 cluster 4 samples (38%) showed a mean 80% dominance of GTX1 only, with trace levels of GTXs (particularly GTX2), STX and NEO.

**Figure 7:**
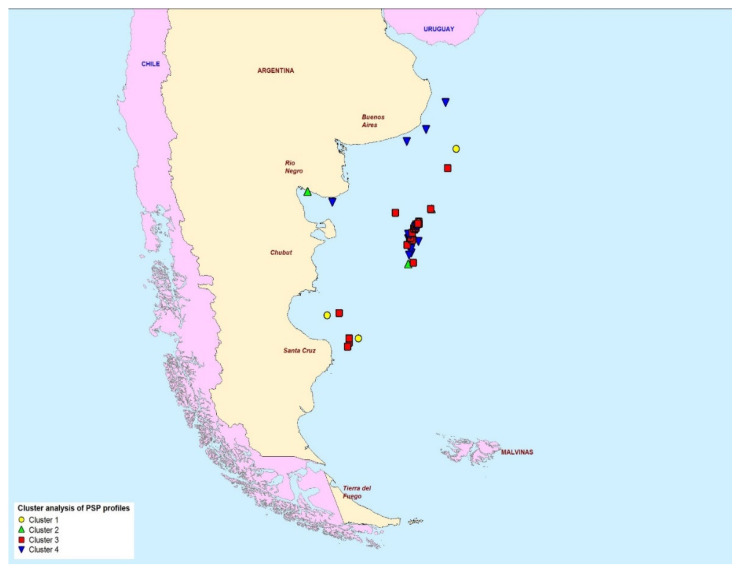
The geographical location of the four paralytic shellfish toxin profile clusters determined in scallops from the Argentine Sea.

Figure 7 maps the geographical location of each of the samples in reference to the profile cluster type. The map shows the presence of cluster 4 profiles around the coastal zones of the northern half of Argentina, although cluster 4 samples were also observed off-shore. Conversely, cluster 3 profiles containing high proportions of GTX1 and GTX2 were found mostly off-shore and in the southern provinces.

**Figure 8:**
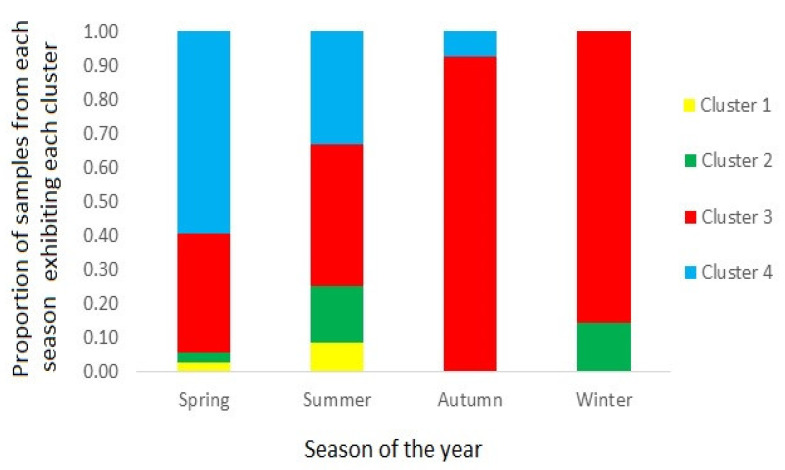
Bar chart showing the proportion of scallop samples from each season showing containing toxin profiles from each of the four clusters.

Figure 8 illustrates the proportion of samples associated with each profile cluster type, in samples harvested during each of the four annual seasons; spring (October to December), summer (January to March), autumn (April to June) and winter (July to September). The results show a near-exclusive dominance of cluster 3 during both the autumn and winter, with increasingly higher numbers of samples associated with cluster 4 during the summer and spring, respectively.

### 2.2. Lipophilic Toxins

Out of 96 whole scallop samples, 95 samples in total (99%) contained detectable levels of regulated LTs, but with all being present at low concentrations (Appendix A; Figure 3). In terms of OA-group toxins, all samples showed levels of toxicity well below the regulatory limit of 160 µg OA eq./kg, reaching a maximum total OA-group concentration of 12.9 µg OA eq/kg. OA-group toxin profiles were dominated by OA, with 12% of the samples containing OA reaching a maximum concentration of 9.5 µg/kg and with only three samples containing trace levels of DTX1. In addition, low concentrations of PTXs (maximum 26.4 µg OA eq./kg) were detected and quantified, with profiles dominated by PTX2; 84% of samples contained PTX2 and averaged 93% of the total concentration in positive samples. PTX1 and PTX11 were found in 12% and 6% of samples, respectively. Other LTs were detected in the samples, such as the Azaspiracids (AZAs) and yessotoxins (YTXs). The maximum total AZA concentration reached 31.4 µg AZA1 eq/kg, approximately 20% of the regulatory action limit of 160 µg/kg. The major AZA analogue detected was AZA2, as opposed to AZA1. Yessotoxins were found to reach a maximum concentration of just over 100 µg/kg, but with a regulatory limit of 3.75 mg/kg, this only represents ~2.5% of the regulatory action level. A total of 19% of the samples contained YTX and 4% contained 45 OH YTX. Yessotoxin profiles comprised mainly of YTX making up to 92% of positive samples. In the majority of processed adductor-only scallops, no LTs were detected, with low numbers of exceptions found in two samples for AZAs (0.7 and 1.1 µg/kg) and in five samples for PTXs (1.1 to 9.5 µg/kg). No YTXs were detected in any of the adductor samples. Spirolides were detected in 92 whole scallop samples, with concentrations reaching a maximum total of 22.9 µg/kg. Low to trace concentrations of spirolides were also detected in some of the adductor muscles, but notably not in the sample containing the highest concentration of spirolides in the whole tissue.

### 2.3. Domoic Acid

Out of all the samples analysed for domoic acid by HPLC-UV, only five were found to contain detectable levels of the toxin. Concentrations were, however, all less than 2 mg/kg, ranging from 0.3 to 1.5 mg/kg. No toxin was detected in any of the adductor samples (Appendix A; Figure 3).

## 3. Discussion

### 3.1. Paralytic Shellfish Toxins

#### 3.1.1. First Study

In the initial study of seven scallop samples, whole *Z. patagonica* scallops were found to contain low concentrations of PSTs, reaching a maximum of 94.5 µg STX eq/kg. Conversely, all three of the whole *A. tehuelchus* samples exhibited PSTs, with total concentrations ranging from 3597 to 14,064 µg STX eq/kg (Table 1). Interestingly, two samples split into both whole tissue and adductor-only sub-samples, showed total toxin concentrations exceeding the MPL of 800 µg STX eq/kg in the adductor and thereby still presenting a food safety threat to consumers of scallop adductors even after processing to remove the non-edible portions of the organisms. The remaining adductor sample showed total PST at 101 µg STX eq/kg, evidencing total toxin concentrations at less than 20% of the regulatory limit. Much lower PST concentrations were quantified in the *Z. patagonica* samples, with all whole tissue samples well below the MPL. Detection of toxins in the adductors from this study is interesting given the general assumption made that the adductor muscles of scallops are safe to eat, even when whole scallops present toxicity above the maximum permitted levels [53]. However, more samples of whole and adductor scallops would be required until it is possible to extrapolate these findings to scallop species in general. Findings of quantifiable levels of PSTs in the adductor muscles of processed scallops would be expected generally at low concentrations, well below regulatory level, assuming the processing is conducted appropriately. Various studies have reported variable, but generally low, concentrations of adductor-based toxins. Ref. [54] showed <3% of total toxins to be stored in the adductor muscles of the noble scallop, *Chlamys nobilis*. Tissue compartments have been shown previously to follow a rank order in terms of toxin load: digestive gland > mantle + gill > gonad > adductor muscle in the scallop *Placopecten magellanicus* [55,56], although, in more recent findings from other scallop species (*Patinopecten yessoensis*), the ordered rank was digestive gland > gonad > mantle > adductor > kidney > gills [57]. Locomotory tissue such as the adductor muscle contributes substantially to the total weight of soft tissues but makes a disproportionately low contribution (<3%) to the toxin body burden [58]. The toxicity of the scallop adductor muscle is typically one to three orders of magnitude lower than that of the corresponding digestive gland and rarely exceeds the regulatory level, even during blooms. Ref. [59] reported that with the digestive glands of the sea scallops, *P. magellanicus,* containing ~120,000 µg STX eq/kg, PSTs remained below the detection limit in the adductor muscles, following testing by MBA. Ref. [60] assessed the accumulation of PSTs in different tissues of juvenile Pacific calico scallops *Argopecten ventricosus*, reporting the highest toxin loads in digestive glands and mantle, with the lowest concentrations in the adductor, kidney and rectum, combined. Evidence points towards the presence of toxins in the adductor but at low concentrations which supports what was found in *Z. patagonica* samples, however, it does not explain why significant concentrations of PSTs were found in the adductor of *A. tehuelchus,* albeit in a low number of samples. This suggests that retention of PSTs in the adductor could be species-specific in scallops, as higher concentrations in the adductor have been observed in previous studies of different scallop species. Ref. [61], for example, reported that between 20% to 48% of scallop toxicity was quantified in the adductors of the raw scallops *P. yessoensis*, with significantly lower proportions of toxins present in the gonad, gill and mantle. In that study, up to 64 µg STX eq. per individual scallop were quantified resulting in a significant risk to consumers of processed adductor muscles [61]. *A*. *tehuelchus* may therefore retain toxins for longer than *Z. patagonica* but due to the small sample size of *A. tehuelchus* in this study, more data would be required to assess this hypothesis appropriately. Other researchers have reported that the highly toxic *P. yessoensis* was found to contain no detectable toxin presence in adductor after processing fresh scallops, whereas adductor samples prepared from frozen scallops were more likely to contain toxins [62]. Whilst the preliminary findings indicated a potential risk from toxins in the adductor muscles of *A. tehuelchus,* in the main study only *Z. patagonica* scallop species were included. Unfortunately, with the prohibition of bivalve commercialization from non-classified areas in 2002 and the consequent lack of availability of *A. tehuelchus* samples from the Buenos Aires region, a systematic comparison of the toxin levels in whole and adductor tissues of this species was not possible.

#### 3.1.2. Main Study Adductor Toxin Load

Twelve percent of *Z. patagonica* adductor muscle samples contained trace amounts of toxins, with the maximum concentration being 71 μg STX eq/kg, representing ~10% of the MPL of 800 μg STX eq/kg. As such, the findings appear to evidence the low risk of PST occurrence in the adductor of the *Z. patagonica* scallop. In terms of processing, the adductor muscle of *Z. patagonica* is obtained very quickly after harvesting of the scallop, as it is processed on board the fishing vessels immediately post-harvest. Therefore, the time between the capture of a live *Z. patagonica* sample and the acquisition of a frozen adductor muscle does not exceed one hour. In *A. tehuelchus,* however, scallop harvesting was carried out by either divers or trawlers, which land their catch after several hours and subsequently process their catch many hours after harvesting [35]. This could imply that the time taken to harvest, process, and the subsequent freezing of these two different species could cause a change in PST content within the different scallop tissue compartments. It has been shown before that fast processing and subsequent freezing on board ships can lead to no detectable levels of PSTs in adductor muscles even when concentrations are high in the whole scallop [63]. Alternatively, the two scallop species could bioaccumulate toxins differently, with different proportions of toxins accumulating in the adductor muscle compared with other tissues, as suggested by the different proportions of toxins determined between scallop tissue compartments in different scallop species [54,56,57,59,63].

#### 3.1.3. Variability in Toxin Concentrations

Variations in scallop PSP toxicity are expected between seasons as a consequence of differing distributions, timings, magnitude, cell toxicities and persistence of source phytoplankton blooms. Variable physiological as well as environmentally-mediated metabolic responses of scallops to algal blooms may also significantly affect toxin uptake, making predictions of scallop toxicity in relation to PST-producing bloom presence, complicated [56]. Certainly, in this study, there is no data describing the proliferation of PST-producing blooms, making the assessment of factors affecting toxin concentrations in scallop tissues challenging. Six of the whole scallop samples contained total PST concentrations greater than the MPL (Table 2). Of these six, five were sampled during the summer months, coinciding with expected high toxin concentrations.

Regions in Argentina follow this trend of higher toxin levels in spring and summer with most toxic blooms generally (but not restricted to) between November and January. The Beagle Channel, since 1992 [64] and Patagonia [65] and Chubut province [66], since 1980, have all exhibited recurring spring and summer PSP toxin levels exceeding the MPL. Recent studies of PSP levels in bivalves from Argentina have demonstrated significant temporal and spatial variability in toxin presence, but with a clear pattern of high toxin concentrations accumulating during spring and summer months [32,66]. In comparison, sample 69 was harvested towards the end of the summer in March 2013, therefore, lower toxicity is expected to be observed in comparison to the summer samples, due to a depletion of nutrients and light levels causing a reduction in microalgal cell density and toxin bioaccumulation in filter-feeding molluscs [67]. However, this was not observed for this sample, which exhibited a total PST concentration of close to 4000 µg STX eq/kg. As such this infers that either PST-producing phytoplankton were still active during this time period and/or potential long depuration times for PST in scallop tissues [55,56,59,68,69]. Different bivalve species are known to exist highly variable depuration rates of PSTs. Whilst mussels are typically associated with fast depuration [58], although not always [65], other bivalves, most notably some species of scallops, may retain toxicity for a year or more [58] with some deep-water scallops transforming the toxins from blooms and storing them from one season to the next [58]. *A. tehuelchus* has been shown to have a lower detoxification rate when compared to other scallop species in the San José Gulf and San Matías Gulf [66]. The *A. tehuelchus* processed in the preliminary study was not continued into the main study, preventing the comparison in detoxification rates between the two Argentinean scallop species across different seasons.

Inter-annual differences were also observed in these data. All six of the *Z. patagonica* samples containing total PST concentrations >MPL were harvested between 2013 and 2014 whereas all samples harvested between 2015–2016 were below this limit. This could be due to a change in bloom dynamics over the years; blooms are not restricted spatially or temporally to a particular area and/or with the same phytoplankton species. Chubut coastal waters have exhibited changing spatial and temporal dynamics of harmful algal species since 1980 [45]. Ref. [32] have also recently confirmed large inter-annual, as well as spatial differences in toxin levels in a variety of bivalve species. Unfortunately, water samples were not provided at the time of scallop harvesting, so it was not possible to clarify whether spatial and temporal differences were related to algal source.

#### 3.1.4. Profiles

PST profiles determined in many of the scallop samples were found to be unusual compared to some of those reported previously in other bivalve mollusc samples from the region [33,70]. The clear dominance in this study of the gonyautoxin analogues GTX1 and GTX2 shows some similarities to the GTX2&3 and GTX1&4 prevalent profiles in mussels and scallops previously described [32], although the latter study utilised Pre-COX LC-FLD analysis which is unable to report concentrations for individual epimers. Specifically, Argentinean mussels harvested throughout the country across all coastal provinces, over a thirty-year time period were shown to contain on average, over 70% GTX1&4 in terms of STX equivalents, with GTX2&3 the other most commonly occurring analogues [32]. Scallops on the other hand were shown to contain a higher proportion of GTX2&3, but still contained significant contributions to overall toxicity from GTX1&4, as well as the presence of STX, C1&2 and lower proportions of decarbamoyl PSTs. The mean profiles for the *Z. patagonica* samples analysed here, therefore showed distinct differences from those determined from the mostly in-shore scallops, published recently [32]. Reports of PST profiles in *P. magellanicus* scallop tissues from Maine, USA, highlighted the dominance of both the low toxicity C1&2 and the higher toxicity GTX1-4, together with NEO and STX, although there were wide variations in profiles, depending on geographical, inter-annual and season of harvest [55,56]. Furthermore, high variabilities in toxin profiles were also reported [57], between different scallop tissue compartments, but still with a high proportion of C toxins, as well as GTX2&3, NEO and STX. Scallops from Hong Kong showed a different PST composition still, containing high proportions of GTX5 along with GTX1-4, NEO, STX, dcSTX and dcGTX2&3 [61], whereas PST profiles in *P. yessoensis* scallops from Japan showed predominantly GTX1-4 inclusive, with only trace levels of C1&2 present [71], as well as the presence of Tetrodotoxin (TTX) which was not detected in any of the samples analysed in this study (data not shown).

Some of the differences between the toxin profiles described here and those from [32] may result from the effects of selective toxin analogue retention and/or toxin transformation which can occur during shellfish metabolism and may be mediated by bacteria and/or enzymes present in the shellfish tissues [10,56,58,72,73,74,75,76,77]. Of particular relevance to the findings in this study are the epimerisation reactions occurring in shellfish during feeding which convert the β-epimers present as the dominant epimers in the dinoflagellates, to an equilibrium of both epimers with the α-epimer dominating the profiles in shellfish [56,75,78,79], but with both epimeric pairs typically present. Therefore, the toxin profiles determined in this study from the tissues of *Z. patagonica* showed interesting differences, given the dominance of the α-epimers GTX1 and GTX2 with very low proportions of the β-epimers GTX4 and GTX3. The dominance of α-epimers could potentially reflect the amount of time these toxins were retained in the scallop tissues after the initial toxin accumulation event, but this is impossible to prove without conducting scallop-feeding studies on various PST-producing phytoplankton species. Epimerisation has been reported in a number of different shellfish species, including scallops, with *Pecten maximus* observed to show epimerisation of C1 and C2 toxins [12,80]. The dominance of α-epimers in scallops from this study, was also observed in Maine scallops, providing further evidence for epimerisation during scallop metabolism [56]. Conversely, it has been highlighted that toxin profiles of *Alexandrium* sp. obtained in the spring were dominated by β-epimers (C2, GTX4, GTX3) whilst in autumn were dominated by α-epimers (C1, GTX1, GTX2), due to epimerisation during unfavourable environmental conditions [81]. This could suggest that the changes to toxin production within the phytoplankton species are potentially responsible rather than *Z. patagonica* and thus the data obtained gives an overview of different populations of phytoplankton at varying temporal and spatial scales. Either way, more work is required to isolate the exact cause(s) of these unusual PST profiles in scallop tissues.

A range of PST-producing microalgal species are known to proliferate through the various coastal regions in the Argentine Sea. The absence of decarbamoyl toxins and the N-sulfocarbamoyl analogues infers that *G. catenatum* is not responsible for toxin accumulation in these scallops, given the dominance of these analogues in *G. catenatum* strains [43]. *Alexandrium catenella* is the microalgal species most commonly linked to high PST concentrations in Argentinean bivalve molluscs. *A. catenella* profiles across the Argentine Sea are typically dominated by C1&2, followed by GTX1&4, as well as Neo and GTX2&3, with an absence of STX [82,83,84], although *Alexandrium* sp. can exhibit highly variable toxin profiles, even within a morphologically homogenous population [85,86]. Consequently, it is likely that the GTX-dominated profiles determined here are related to a source *Alexandrium* species, with the addition of epimerisation resulting in the dominance of GTX1 and GTX2.

Cluster analysis of the PST toxin profiles determined by LC-MS/MS evidenced the existence of separate profile type groups, with the majority of samples falling into either cluster 3 (high proportions of GTX1 and GTX2 combined) or cluster 4 (dominance of GTX1 only) (Figure 6). Previous work has highlighted the potential for toxicity differences to arise between scallops harvested from coastal and deep-water environments [56,58,68], although these differences were not assessed in this study given the majority of scallops originated from the deeper waters, hundreds of miles east off the coast of Chubut Province. Profile types were plotted on a map, but there was no clear visual evidence for any geographically-related factors which may affect the toxin profiles within the scallop tissues, with the main group of centrally-located samples consisting of scallops with both cluster 3 and 4 profiles (Figure 7, inset).

The majority of samples associated with cluster 4, containing a dominance of GTX1, were harvested between October and March which coincides with the spring and summer seasons and thus when PST-producing microalgal blooms are most prolific [64,66]. Spring and summer samples were also associated with the cluster 3 profile, containing both GTX1 and GTX2 analogues. Scallops harvested during the autumn and winter seasons were associated almost exclusively with cluster 3 profiles (Figure 8), although this may relate to the lower number of samples harvested in these seasons. A recent study of PST profiles in bivalve molluscs throughout Argentina, also showed the dominance of GTX1&4 epimers during the spring season, although also during the winter, with GTX2&3 the next most prevalent analogues, with significant proportions of both epimeric pairs still present during the remaining seasons [32]. However, that study utilised PreCOX LC-FLD analysis for toxin quantitation, so was unable to confirm the proportions of epimers present, for either GTX1&4 or GTX2&3. Overall, the changes in profiles through the seasons, confirm similar findings for significant seasonal fluctuations reported previously in scallops (e.g., [55,56]).

### 3.2. Lipophilic Toxins

The majority of scallop tissues in this study were found to contain detectable levels of the spirolide, SPX1, present in ~94% of whole tissue samples with a mean concentration of 8.9 µg/kg and reaching a maximum of 22.9 µg/kg (Table A1). SPX1 has been reported previously in the marine environment around Argentina, both in shellfish and seawater at low concentrations [37,43,84,87]. SPX1 has been shown to be produced from *Alexandrium ostenfeldii* in a wide proportion of Argentinian waters [43,85,87], as well as other spirolides not tested in this study, so findings of low concentrations of SPX1 in these samples were not unexpected.

Of the three pectenotoxins incorporated into the testing method (PTX1, PTX11 and PTX2), the most prevalent was PTX2 being detectable in 75% of whole scallop samples with a mean concentration of 7.6 µg/kg and a maximum concentration of 28.3 µg/kg (Table 3). PTX2 has been detected previously in phytoplankton samples in Argentina [33,70,83,85,88] and was found to be the most dominant toxin in geographic distribution and abundance [84]. PTX2 has been discovered across the entire Argentine sea previously with PTX2 presence associated with *Dinophysis tripos* [85,87] which can be found in shelf waters between ~36–55 °S [46]. In 2015 samples of *D. tripos* were found to produce PTX11 and PTX2-seco acid in small concentrations [89]. Ref. [37] also reported the first findings of PTX2 in Argentinean bivalves, including scallops, mussels and clams, although all concentrations were below 16 µg/kg.

Out of the three AZA toxins tested (AZA1-3), AZA 2 was the most dominant in 30% of the samples with an average toxicity of 2.41 µg/kg and a maximum concentration of 31 µg/kg, 20% of the MPL (Table A1). Consequently, the AZA analogue detected in the scallops from this study confirmed the previous findings of AZA2 which was first reported in Argentinian mussels and clams in 2015 at low concentrations [37]. In terms of AZA microalgal producers, species known to inhabit Argentinian waters include *Azadinium poporum*, *A. dexteroporum*, *A. spinosum* and *Amphidoma languida* [33]. *A. spinosum* is the likely cause of this toxication due to the previous detection of AZA2 in Argentinean bivalves [47].

Of the four YTX analogues incorporated into the LC-MS/MS method, two were detected, YTX and 45OH-YTX with 35% of whole scallop samples containing YTXs. YTX had a mean concentration of 35.3 µg/kg in whole tissues and a maximum concentration of 68.1 µg/kg, thereby showing concentrations at levels <2% of the MPL of 3.75 mg/kg (Table A1). Interestingly, during a previous study of LTs in Argentinean bivalves, YTX was only detected and quantified in scallop samples, with no YTXs in mussels or clams [37]. YTX has also been reported in Argentinian waters in the dinoflagellate *Protoceratium reticulatum* [48].

Whilst OA-group toxins such as OA and DTXs have previously been found at high concentrations in Argentinean bivalves, these were almost exclusively mussels and clams, with only one scallop sample found previously to contain 45.6 µg/kg total OA eq/kg [37]. In this study, 26% of whole scallop samples were found to contain OA-group toxins, but at total concentrations <5% of the MPL of 160 µg/kg, with a maximum of 6.2 µg/kg (Table A1). In terms of OA-group toxin profiles, OA was the dominant analogue, with DTX1 only detected in four samples, with a maximum concentration of 12.5 µg/kg. No DTX1 was detected in any of the scallop tissues. Both OA and DTX1 have been previously reported in Argentinian waters [33] albeit in much higher concentrations. Previous studies have reported the presence of *Dinophysis* species associated with DSP toxin production, including *D. acuminata, D. caudata, D. tripos, D. fortii* and *D. norvegica* [33,85,87]. Additionally, *D. acuminata* and *D. rotundata* are known producers of DTX1 and have previously been discovered in the gut of *Z. patagonica* highlighting the potential for the observed OA and DTX1 concentrations in this study. Overall, however, given the previous DSP outbreaks and human intoxications following consumption of bivalves contaminated with OA-group toxins [36,37,83,90,91], the evidence from this study indicates a low level of risk from DSP in whole scallop tissues.

Whilst LT concentrations were generally low in the whole scallop tissues, concentrations and detection rates were even lower in the adductor muscle samples. PTX2 was detected in 75% of the whole tissue samples as opposed to 7.7% of the adductor samples, with only 5.5% of adductor samples containing AZA2 in comparison to >50% of the whole tissue samples. Similarly, low detection rates were found for OA-group toxins (1.1% of samples) and YTXs (2.2% of samples). Conversely, >50% of adductor samples were still found to contain detectable levels of SPX1, albeit at low concentrations (mean 2.4 µg/kg). It is also noted that whilst only low concentrations of spirolides were quantified, the method did not incorporate the detection of pinnatoxins, due to the lack of standard availability at the time of testing. These toxins have recently been detected in a range of shellfish species, as well as causing outbreaks in humans following direct exposure to *Vulcanodinium regosum* in seawater [92,93,94]. Future work should also ideally assess the presence of pinnatoxins and quantify the potential risks from seafood consumption. Overall, therefore, whilst the toxin concentrations present in the whole tissues were already well below MPLs, concentrations in processed adductor muscle samples were a lot lower, indicating that during events when higher toxin concentrations may accumulate in scallops, the risk to human health should still be reduced when scallops are processed properly. Further testing would be required to assess this, if/when toxins are ever found at higher concentrations in whole scallop tissues.

### 3.3. Domoic Acid

Results from this study have illustrated the low prevalence and toxicity of DA in *Z. patagonica* scallop species in the Argentinian sea, with only five whole scallop samples containing detectable levels, all of which being <2 mg/kg. In regard to the MPL of 20 mg/kg, these five samples would not pose a risk to human health. *Z. patagonica* has previously shown to show no apparent relationship with DA-producing diatoms *Pseudo-nitzschia australis* and *Pseudo-nitzschia multiseries* during periods of blooming [95], displaying no detectable levels of DA despite the favourable environmental conditions that would in general lead to a high concentration of DA in tissues. This would insinuate that *Z. patagonica* has a low prevalence for either DA-producing phytoplankton, a very rapid excretion of toxins from tissues or there is a dominance of non-toxigenic phytoplankton.

The low prevalence of DA indicates the low impact of *Pseudo-nitzschia* species on scallops within offshore beds within the Argentine Sea. Occasional and low-level bioaccumulation of DA might be related to the time and location of harvesting. Certain *Pseudo-nitzschia* species that produce DA such as *P. pungens* or *P. seriata* were noted to dominate waters with colder temperatures (4–20 °C) [96,97]. The samples in this study were mostly harvested during the summer season which could be outside the time period when dense toxigenic blooms were proliferating. The geographical location of scallop beds may also be a factor, with shallow water regions associated with limited circulations providing the conditions of stress and excess inorganic nitrogen needed for DA production [97]. The fact that the scallops were harvested offshore in water depths between 65 m and 120 m could explain the absence of DA. Though the samples were generally free from DA, the appearance of toxigenic *Pseudo-nitzschia* species and the presence of DA have been confirmed along the coastline of Argentina as far back as the 1930s [33]. Nevertheless, Argentina has not suffered from human ASP outbreaks in the past due to the successful routine official control monitoring, utilising phytoplankton taxonomy and knowledge on bloom dynamics [33] but also noting that regulatory analysis results collected to date showing no evidence for DA-positive bivalve samples, even in the presence of toxigenic *Pseudo-nitzschia* species.

## 4. Conclusions

This study reports the findings following the analysis of scallops harvested from offshore benthic environments within the Argentine sea for the presence of marine biotoxins. Results highlighted that PSTs are the most prevalent toxins detected in *Z. patagonica* scallops. The low toxicity in *Z. patagonica* adductor muscles, relative to the whole animal samples, confirms the results from previous studies of other scallop species in other geographical regions, which suggest that adductor muscles of scallops can generally be considered safe for human consumption even if the other organs might contain toxins, assuming scallop processing is conducted safely and following strict guidelines. The preliminary study incorporating a low number of *A. tehuelchus* samples did show high concentrations of PSTs remaining in the adductor, however, so further work would be required on this species before adductor processing is used as a sole mechanism for toxicity control in this species. Levels of toxicity in *Z. patagonica* were examined in relation to temporal and geographic factors. Geographically, no significant conclusions could have been drawn, however, samples harvested between 2013–2014 only were found to contain PSTs at concentrations above the MPL which could hint towards temporal (yearly) changes in bloom dynamics or phytoplankton species present. The toxin profiles were dominated by gonyautoxins (GTX1&2) representing up to 87% of the total STX equivalents inferring biotransformation including epimerisation of microalgal toxins during shellfish feeding. LT analysis showed the presence of low concentrations of OA-group toxins including OA, DTX1 and PTXs. In addition, AZAs, SPX1 and YTX were identified, but all at levels below the regulatory action limit. HPLC-UV analyses indicated most samples were free of DA, a direct result of deep water harvesting conditions which do not provide suitable conditions to support phytoplankton species and thus domoic acid production. Overall, whilst some toxins can reach dangerous concentrations in whole tissues, effective processing should enable these products to still provide a healthy and safe product for human consumption.

## 5. Materials and Methods

### 5.1. Samples

Two different scallop species *A. tehuelchus* and *Z. patagonica* were analysed in the initial study. Seven scallop sample homogenates were collected from different regions of the Argentine sea between 1986 and 2012 as part of the routine toxin monitoring program. These were used as a preliminary assessment of PST presence in the whole tissues and adductor of scallops. Four of the whole scallop samples have been analysed previously for PST and reported [32] but were incorporated into the preliminary assessment in this study in order to compare PST levels in whole vs adductor tissues. Following these results, a larger sampling study was conducted, with the collection of 96 *Z. patagonica* samples from different regions of the Argentine Sea, as part of the routine monitoring program between 2012 and 2017. There was a total of 82 individual sampling locations which provide a spread of data along the east coast of Argentina. No *A. tehuelchus* samples could be incorporated into the main study as the species was no longer collected commercially from in-shore coastal waters of the Buenos Aires Province. Figure 2 maps the location of all sampling points incorporated into this study.

*A. tehuelchus* samples taken prior to 2012 comprised whole samples collected by commercial fishing vessels. For each sample, approximately 20–30 animals were collected and stored refrigerated until they were sent to the testing laboratory. At the lab, whole scallop homogenates were tested by MBA. Leftover whole scallops were also processed and the adductor muscles also tested for PSP toxicity. One exception was sample 110C, which was received only as frozen adductor muscle from *A. tehuelchus* which was processed on land and exhibited PSP toxicity by MBA. *Z. patagonica* samples were taken by government officials on board scallop factory trawler vessels. After the harvesting area location was recorded, officials sampled approximately 1 kg of live scallops, normally equating to 25–30 animals. Harvested scallops were immediately processed on board to remove the shells and all soft tissues, apart from adductor muscles. Adductors were then quickly frozen and a 500 g sub-sample of the adductor was taken. Official samples of both whole scallops and processed adductor muscles were then stored frozen on board until the vessel returned to port, and the samples were shipped to official control laboratories. Frozen subsamples were later transported to the Cefas laboratory under temperature-controlled conditions where upon reception they were temperature checked (<−16 °C) and subsequently stored frozen until required for analysis. In terms of biosafety, shellfish samples were transported frozen and held frozen throughout the shipment. Once extracted and analysed remaining tissues and extracts were disposed of through incineration at a licenced establishment.

### 5.2. Methods of Analysis

#### 5.2.1. Reagents and Chemicals

All solvents, reagents and chemicals were of LC-MS or HPLC grade, depending on the system-specific requirements. A MilliQ water purification system (Merck, Darmstadt, Germany) was used to provide LC-MS grade water used for preparing mobile phases. Certified reference material (CRM) standards for all analytes were obtained from the Institute of Biotoxin Metrology, National Research Council Canada (NRCC, Halifax, Nova Scotia, Canada). PSTs incorporated were GTX1-6, dcGTX2&3, dcSTX, dcNEO, NEO, STX and C1&2. Non-certified toxin standards were also received from Cawthron Natural Compounds (CNC; Nelson, New Zealand) for C3&4 and dcGTX1&4. LT analogues incorporated included OA, DTXs, PTXs, YTXs, AZAs, GYM and SPXs. A domoic acid standard was utilised for the quantitation of DA. Matrix reference materials used for quality control included an oyster matrix CRM for PSP toxins (Cefas, Weymouth, UK), a freeze-dried mussel tissue for LT and mussel matrix CRM for domoic acid (NRCC).

#### 5.2.2. PST Analysis

Samples were analysed utilising ultra-high-performance liquid chromatography with tandem mass spectrometry (UHPLC-MS/MS) based on [98]. A single-step dispersive extraction was used with 5 g aliquots of homogenised shellfish tissue in each sample extracted with 5 mL of 1% acetic acid following [18,57,99]. For internal quality control (IQC) purposes samples were extracted in batches alongside a negative control procedural blank (PB), a positive control laboratory reference material (LRM) and a matrix CRM. Crude acidic scallop extracts were cleaned using an automated solid phase extraction (SPE) (Gilson; Dunstable, Bedfordshire, UK) ASPEC XL4 system. Samples underwent graphite SPE cleanup to remove salts from the extract [57]. A total of 100 µL of SPE eluents were subsequently diluted with 300 mL of LC-MS grade acetonitrile prior to analysis.

LC-MS/MS analysis was performed using an Agilent (Manchester, UK) 6495B triple quadrupole tandem mass spectrometer, coupled to an Agilent 1290 Infinity II UHPLC system for chromatographic separation. Nineteen PSTs were quantified using a 6-point calibration curve prepared in diluted, SPE-cleaned mussel matrix for each of the following calibrants: STX, NEO, dcSTX, dcNEO, doSTX, GTX2, GTX3, GTX1, GTX4, GTX5, GTX6, dcGTX2, dcGTX3, dcGTX1, dcGTX4, C1, C2, C3 and C4. Chromatographic separation was performed using a Waters (Elstree, Herefordshire, UK) Acquity BEH Amide (1.7 µm × 2.1 mm × 150 mm) column in conjunction with a Waters BEH Amide guard cartridge. Parameters for UHPLC gradient solvent delivery, mass spectrometer source conditions and MRM transitions are those published previously [100]. Example MRM chromatograms are shown in Supplementary Materials Figure S1. Measurement uncertainties associated with the method are summarised in [99]. LODs for the validated method range from 0.1 to 7 µg STX eq/kg (mean = 1.4 µg STX eq/kg) [99].

#### 5.2.3. LT Analysis

Samples were analysed utilising reverse phase LC-MS/MS. A double-step methanolic extraction based on the EURLMB protocol [31] was used, 2 g aliquots of each homogenised sample were extracted in 9 mL methanol. The supernatants were collected, and the remaining pellets were subjected to a second extraction step whereby 9 mL of methanol was added, and then homogenised with IKA Ultra Turrax homogenisers (Oxford, Oxfordshire, UK). The supernatants were combined and diluted to 20 mL with methanol and then filtered using 0.2µm nylon filters (Phenomenex, Manchester, UK). For IQC purposes samples were again extracted in batches alongside a PB, LRM and CRM. Methanolic extracts from all samples and controls were hydrolysed to allow the determination of acyl esters of OA-group toxins [31].

Both hydrolysed and unhydrolysed extracts were analysed using two UHPLC systems, Acquity and Acquity I-class which were coupled to a Xevo TQ and Xevo TQ-S triple quadrupole mass spectrometer, respectively (Waters Ltd., Elstree, Herefordshire, UK). Chromatographic separation was conducted using an Acquity BEH C18 (1.7 µm × 2.1 mm × 50 mm) column with a Waters BEH C18 guard cartridge. UHPLC and MS/MS conditions utilised were exactly those described [101,102]. Example MRM chromatograms are provided in Supplementary Materials Figure S2. This method quantified the toxins OA, DTX1-3, PTX1,2,11, YTX, h. YTX, 45 OH YTX, 45 OH h. YTX, AZA1-3, GYM and SPX1 using a 6-point calibration curve prepared using certified calibrants. Toxin concentrations were summed for each group (OA-group, AZAs, YTXs) and converted to total equivalents using standardised TEFs. Measurement uncertainties associated with the method are summarised in [101,102]. LODs for the method are 1 µg/kg for all LT analytes based on current and validated instrument performance.

#### 5.2.4. Domoic Acid Analysis

Two grams of aliquots of homogenate were extracted with 18 mL of 50% methanol with homogenisation using IKA Ultra Turrax homogenisers (Oxford, Oxfordshire, UK) as described by [103]. After centrifugation at 4000 rpm for 10 min, methanolic extracts were filtered using 0.22 µm nylon filters. The filtered samples were analysed by LC-UV using Agilent 1100 LC system (Agilent, Manchester, UK) consisting of a quaternary pump, Diode array detector (DAD), vacuum de-gasser, autosampler and thermostatically controlled column oven. Chromatographic separation was achieved using a Phenomenex C18 (5 µm × 4.6 mm × 150 mm) column (Phenomenex, Manchester, UK) with a solvent gradient. This method quantified domoic acid and epi-domoic acid, with quantitation achieved using a 4-point calibration curve prepared using dilutions of certified calibrant standards. An example LC-UV chromatogram is shown in Supplementary Materials Figure S3. Measurement uncertainties associated with the method are summarised in [103]. The LOD for DA analysis is 0.1 mg/kg. 

### 5.3. Data Assessment

Prior to this study analysis for PSP by MBA at Senasa, had previously shown positive results for several scallop adductor muscle samples as well as in whole scallop tissues (data not shown). Consequently, a preliminary assessment was made here, with the analysis of seven scallop samples from two species, with LC-MS/MS quantitation of PSTs. These data were used to assess any preliminary evidence for risks from PST accumulation in scallops and the potential presence of toxins in edible tissues post-scallop processing. Following this assessment, a larger number of scallops were extracted and analysed for all three toxin classes. In addition to determining total toxin concentrations and associated toxin profiles, the potential spatial (geographical location) and temporal (year of harvest) effects were examined. K-means cluster analysis [52,86] was used to assess the presence of any specific profile patterns for PSTs not identified through any of the above analyses, utilising a minimum total toxicity cut-off of 20 µg STX eq/kg. This process was conducted by developing algorithms in Excel, applying them to the relative proportions of each PST analogue, to statistically partition all observations into specific clusters of related toxin profiles, minimising within-cluster variances. Cluster centres were generated and applied to all analogues across all relevant results, confirming through the iterative generation of minimum distances to each centre the presence of four distinct clusters.

## Figures and Tables

**Table 1:** Scallop samples analysed in the preliminary study and their corresponding paralytic shellfish toxin toxicity.

Scallop Species	Date Harvested	Lat South/Long West	Part	Total PST (μg STX eq/kg)
*A. tehuelchus*	3 October 1986	38°50′/58°30′	Whole	14,064
			Adductor	854
*A. tehuelchus*	28 October 1986	41°20′/63°20′	Whole ^†^	4865
			Adductor	1289
*A. tehuelchus*	17 October 2000	38°20′/57°20′	Whole ^†^	3597
			Adductor	101
*A. tehuelchus*	2 January 2006	40°56′/64°33′	Adductor	0.5
*Z. patagonica*	20 October 2009	47°00′/64°00′	Whole	94.5
*Z. patagonica*	14 June 2011	46°02′/65°22′	Whole ^†^	185
			Adductor	1.8
*Z. patagonica*	27 January 2012	41°41′/58°08′	Whole ^†^	nd
			Adductor	nd

† Whole scallop samples with toxin data published previously [32], included to show comparison with adductor scallop samples from this study.

**Table 2:** The six whole scallop samples containing total paralytic shellfish toxin concentrations greater than the Maximum Permitted Limit, showing toxin profile cluster number.

Sample Number	Date Harvested	Concentration (μg STX eq/kg)	Cluster Number
69v	4 March 2013	3782	3
75v	13 November 2014	3390	3
70v	16 October 2014	2224	3
73v	21 November 2014	2028	1
71v	12 October 2014	1604	3
74v	1 November 2014	1010	4

**Table 3:** Summary of percentage whole and processed scallop samples containing detectable levels of selected lipophilic toxins and their mean concentrations (µg/kg).

Tissue	OA/DTXs	PTXs	AZAs	YTXs	SPX
Whole Scallop (V)	26.0%	75.0%	58.3%	35.4%	93.8%
Adductor Only (C)	1.1%	7.7%	5.5%	2.2%	57.1%
Mean concentration V (µg/kg)	5.4	7.6	1.9	35.3	8.9
Mean concentration C (µg/kg)	5.6	2.7	12.1	19.9	2.3

## Data Availability

Data is contained within the article and Supplementary Materials and Appendix A.

## Supplementary Materials

The following supporting information can be downloaded at: https://www.mdpi.com/article/10.3390/md20100634/s1, Figure S1: Example MRM chromatograms illustrating chromatographic separation and detection of PST analogues from a mid-level calibration standard prepared in shellfish extract; Figure S2: Example MRM chromatograms illustrating chromatographic separation and detection of LT analogues from a mid-level calibration standard prepared in shellfish extract; Figure S3: LC-UV chromatograms showing evidence of domoic acid in positive laboratory reference material. i—Matrix peaks ii—Domoic acid iii—epi-domoic acid.

## Appendix A

Summary of paralytic shellfish toxins (µg STX eq/kg), okadaic acid-group (µg OA eq/kg), azaspiracids (AZA-1 eq/kg), pectenotoxins (µg/kg), yessotoxins (µg YTX eq/kg) and Domoic acid (mg/kg) concentrations determined in whole *Z. patagonica* scallops, displayed in chronological order, with concentrations quantified in adductor only samples shown in parentheses, also showing date of harvest and known geographical location in terms of coordinates.

**Table A1:** Summary of samples analysed, date harvested, geographical location and total toxin concentrations; total OA-group (µg OA eq/kg), total PTXs (summed PTXs in µg/kg), total µg AZA1 eq/kg; total µg YTX eq/kg, sum SPXs µg/kg; DA/epi-DA mg/kg; total PSTs µg STX eq/kg.

Sample Number	Date Harvested	Lat South/Long West	Total OA Group	Total PTXs	Total AZAs	Total YTXs	SPXs	DA + Epi DA	Total PSTs
67	20 May 2012	47°20′/64°45′				63.4	2.9		774
68	22 May 2012	45°56′/64°39′	3.7	1.7		101.1			472
69	4 March 2013	41°46′/60°01′		3.1		75.1	2.4		3782
70	16 September 2014	46°59′/64°31′		19.2	0.1	76.5			2224
71	12 October 2014	43°50′/59°44′	7.6	24.2		72.2			1604
74	1 November 2014	42°19′/58°59′	12.9	0.7	0.9	67.5	10.9		1010
72	5 November 2014	42°19′/59°04′	5.2		3.3		3.5		93.7
75	13 November2014	42°15′/59°03′	5.9	1.6	0.7	37	15.4		3390
73	21 November 2014	42°11′/58°52′	5.1	3.8	2.8	34.6	20		2028
66	8 November 2015	42°22′/59°03′		30.2 (2.6)	3.8 (1.1)		12.6 (2.0)		
65	21 Januar 2015	42°20′/59°02′		8.2 (1.7)		12	11.2		356
81	16 Februar 2015	42°20′/59°06′	3.8	1.7	0.5	32.2	8.8		325 (28.5)
1	11 May 2015	42°28′/59°12′		4.2	3.8	24.8	9.6		189 (0.9)
105	11 May 2015	42°28′/59°12′		1.7	3.8		9.1		113
50	16 May 2015	42°14′/58°55′	5.1	1.2	4.9	28.7	22.9		209 (0.9)
104	16 May 2015	42°14′/58°55′		2.2		10.3	21.6		261
2	18 May 2015	42°26′/59°13′		0.6	3.4	26.1	9.3 (1.9)		208
101	18 May 2015	42°26′/59°13′		1.8	3.6		7.9 (0.5)		245 (0.5)
3	25 May 2015	42°25′/59°13′	3.5		3.7	52.9	13.4 (1.8)		280
102	25 May 2015	42°25′/59°13′	3.7	2.1	3.8		7.9 (0.5)		306 (0.7)
4	27 May 2015	42°20′/59°09′	3.6		3.6	26.9	11.7		194
103	27 May 2015	42°20′/59°09′		1.5	4.1	13.2	14.1		391 (0.3)
26	4 June 2015	42°24′/59°06′			0.9	28.3	12.2		160 (70.6)
84	2 July 2015	42°13′/58°56′	4.8	1.7		37.3	20.1		237
89	25 July 2015	39°56′/56°35′		1.4	1	34.2	15.8 (2.3)		233
5	15 August 2015	40°05′/56°20′			3		3.7 (1.8)		
6	16 August 2015	41°37′/58°02′				26.5	10.4 (1.8)		79
7	17 August 2015	42°14′/58°52′			3.1		12.9 (2)		102 (0.9)
8	19 August 2015	43°06′/59°49′	3.4		3		16.1 (2.4)		101
9	23 August 2015	43°43′/60°02′					4.0 (2.0)		
11	3 September 2015	43°56′/60°02′			3.2		3.5 (1.8)		
12	4 September 2015	43°13′/59°17′			3.2 (0.7)		2.1 (1.8)		93.2
14	11 October 2015	41°39′/58°00′			3.3		6.1 (1.6)	1.52	27.1
27	3 November 2015	43°59′/60°08′	5.8				6.1		
28	5 November 2015	43°36′/59°35′			0.4		5.1 (5)		
29	12 November 2015	43°17′/59°22′			0.4		5.3		
30	18 November 2015	43°10′/59°21′	5.8		0.5		5.5		
51	21 November 2015	42°08′/58°51′		14.3	2.7	32.2	21.4 (1.7)		108
17	22 November 2015	45°03′/60°15′		0.6	0.5		5.6		
31	26 November 2015	43°20′/59°28′		0.4	0.3		5.3		
32	27 November 2015	43°32′/59°33′		1.7	0.3		5.1		
19	11 December 2015	45°00′/60°12′		13.2			5.3	1.53	
52	14 December 2015	42°15′/58°59′		4.8			12.3 (1.6)	0.36	95.1
53	20 December 2015	42°16′/59°01′		1.3	2.6		10.2 (1.8)		97.7
33	21 December 2015	42°03′/58°17′		5.7	0.5		7.7		
20	22 December 2015	44°52′/60°06′		1	0.3				
34	22 December 2015	43°20′/59°28′		3.3	0.4		5.7		
54	27 December 2015	42°17′/58°54′		3.4			12.3 (1.9)		51.1
21	29 December 2015	45°02′/60°12′	3.2	6.2			5.2		
35	29 December 2015	43°24′/59°37′		2.7			5.7		
36	1 January 2016	42°52′/59°26′		1.4	0.5		12.1		61.2
56	1 January 2016	42°39′/58°54′		11.2 (9.5)	2.3	20	7.2 (2.4)		2.8
57	4 January 2016	39°42′/56°13′		1.1	22.5		2		3.5 (2.0)
37	6 January 2016	45°04′/60°17′		2.1	0.3		5.3		
62	11 January 2016	37°14′/56°00′		5.4	2.5	26.5	15.4		44.5
58	12 January 2016	39°39′/56°09′		3.1		16.5	11.3 (2.1)		4.8 (2.1)
38	17 January 2016	45°06′/60°19′		14.5	0.5		6		
39	22 January 2016	42°50′/59°24′			0.4		10.7 (5)		25.3
40	25 January 2016	42°52′/59°35′			0.7		9 (5.1)		71.1
64	26 January 2016	39°09′/55°55′		2.2 (1.1)	2.6	14.8	13.8 (2.1)		14.6
90	1 February 2016	42°49′/59°32′		2.2			8.9 (2.1)		54.5
41	8 February 2016	42°39′/59°28′		4.8	0.6	26.5	10.6 (5)		28.9
42	13 February 2016	42°50′/59°31′		19	2.4		8.2		123
43	15 February 2016	43°18′/59°37′		25.2			3.3		
44	16 February 2016	44°42′/60°09′		12.4	2.4		1.7		
45	23 February 2016	44°43′/60°05′		9.7			1.6		6.2 (1.5)
59	28 February 2016	39°05′/55°50′		2.4		20.6	9.6 (2.0)		3.1
46	1 March 2016	44°11′/60°33′	3.9	25.3	2.5		3.3		80.9
47	2 March 2016	44°34′/59°41′		14.4	2.4		2.1		3.4
22	9 March 2016	39°17′/55°58′			0.4	35.2	14.1 (5.2)		
85	25 April 2016	43°27′/59°44′		7.8	0.3		4.6		1.3
88	28 April 2016	43°44′/59°48′		5.8			2.5		
80	06 May 2016	43°43′/59°49′		2					3.5
86	15 September 2016	43°14′/59°38′		1.3			8 (1.9)		52.6
23	16 September 2016	39°04′/55°53′				26.5	11.9 (5.1)		
60	20 September 2016	43°55′/59°49′		1	31.4	19.7	4.5		2.2
83	22 September 2016	42°41′/59°29′		1.4			7.3		51.6
61	13 October 2016	43°26′/59°47′		1			2.1		2.5
87	20 October 2016	42°39′/59°36′	5.1	1.4		32.9	7.5 (2.1)		70.7
25	28 October 2016	42°36′/59°23′	5.2		0.4	23.7	14.8	1.32	87.2
93	5 November 2016	42°49′/59°36′	6.2	7.7	0.3		10.7 (1.8)		234 (67.9)
94	6 November 2016	42°47/59°35		8	3.4		13		404
95	7 November 2016	42°48′/59°36′		8.7		6.3	11.8 (0.5)		207 (35.8)
96	8 November 2016	42°47′/59°34′	4.5	11.2			10.4		235
97	9 November 2016	42°48′/59°33′		12.4	3.5		11.4 (0.5)		332 (36.1)
98	10 November 2016	42°48′/59°32′		10	3.5		12.4 (0.6)		464 (36.4)
99	11 November 2016	42°50′/59°40′		20.5			8.5		437 (36.0)
48	12 November 2016	43°25′/59°41′	4.1				2.7		548 (111)
82	13 November 2016	42°40′/59°27′	5.9	25.2			11.4	0.37	50 (36.3)
100	14 November 2016	42°57′/59°09′		23.3			8.6		399
24	16 November 2016	47°10′/64°36′		26.4		24.7	6.7 (5.2)		646
49	21 November 2016	43°31′/59°51′		11.4			3.5		662
79	23 November 2016	42°46′/59°33′	4.1	7.1	0.6		6.6		324 (28.6)
78	3 December 2016	42°45′/59°33′		9.5	0.3	31.6	6.7		169 (28.4)
92	19 December 2016	42°54′/59°28′	12.5	18.9 (1.9)			10.1 (1.8)		293
91	16 March 2017	38°57′/55°46′		1.7			5.8 (1.8)		1.4
