# Supplementary material for: Marine Biotoxins in Whole and Processed Scallops from the Argentine Sea
Source: Mar Drugs. 2022 Oct 10;20(10):634. doi: 10.3390/md20100634 (PMC9604692; doi:10.3390/md20100634)

Supplementary Figure S1

Example MRM chromatograms illustrating chromatographic separation and detection of PST analogues from a mid-level calibration standard prepared in shellfish extract

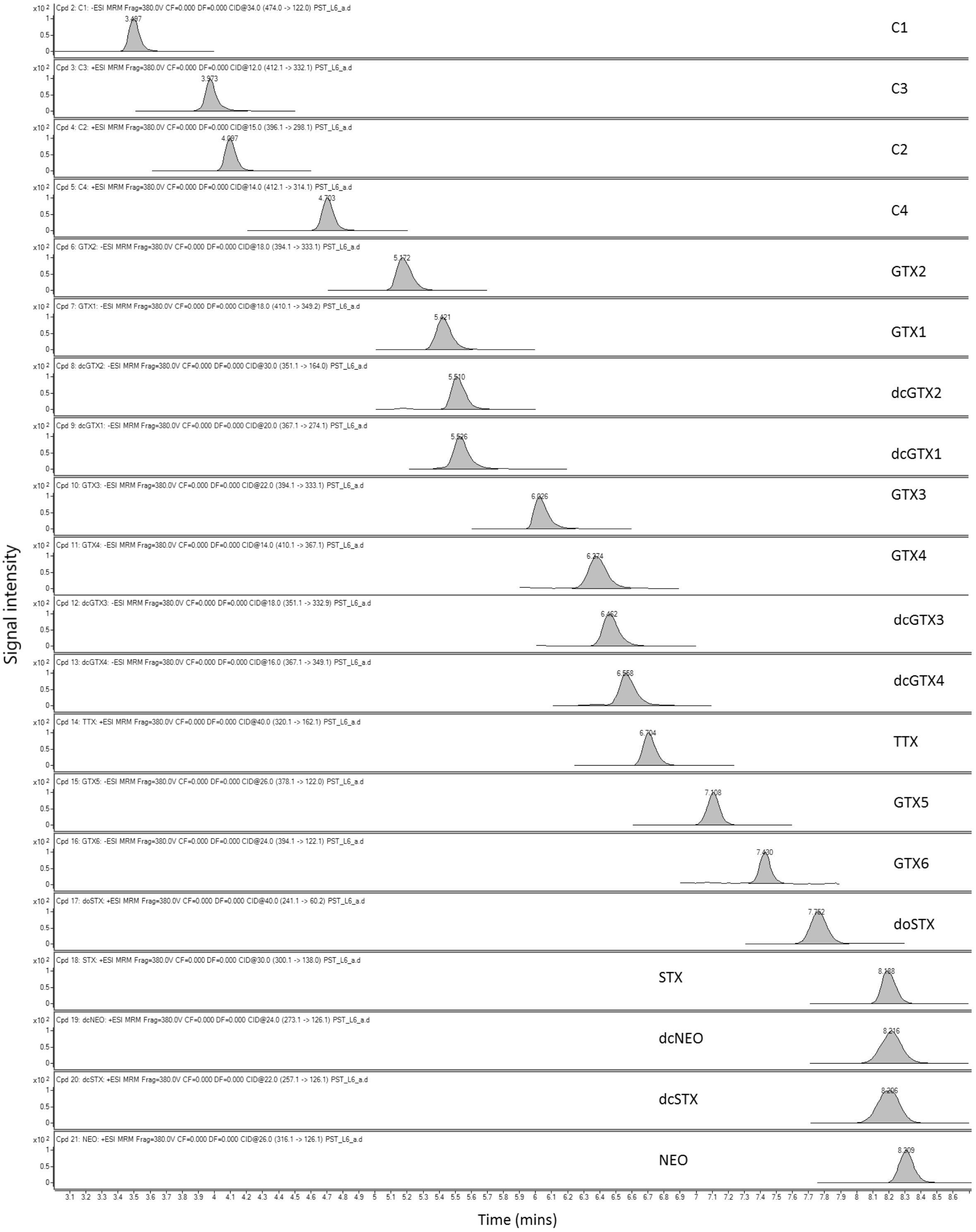

Supplementary Figure S2

Example MRM chromatograms illustrating chromatographic separation and detection of LT analogues from a mid-level calibration standard prepared in shellfish extract

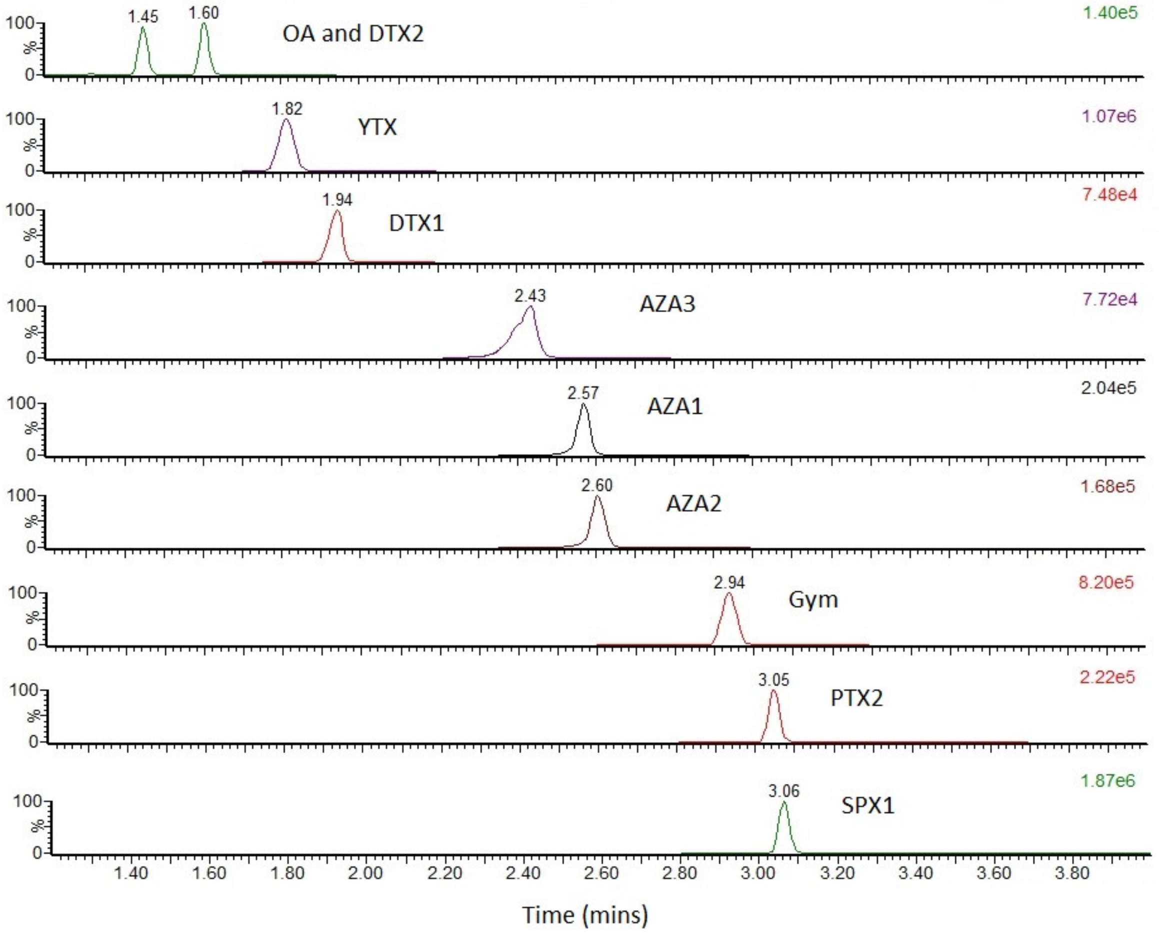

Figure S3. LC-UV chromatograms showing evidence of domoic acid in positive laboratory reference material. i – Matrix peaks ii – Domoic acid iii – epi-domoic acid.

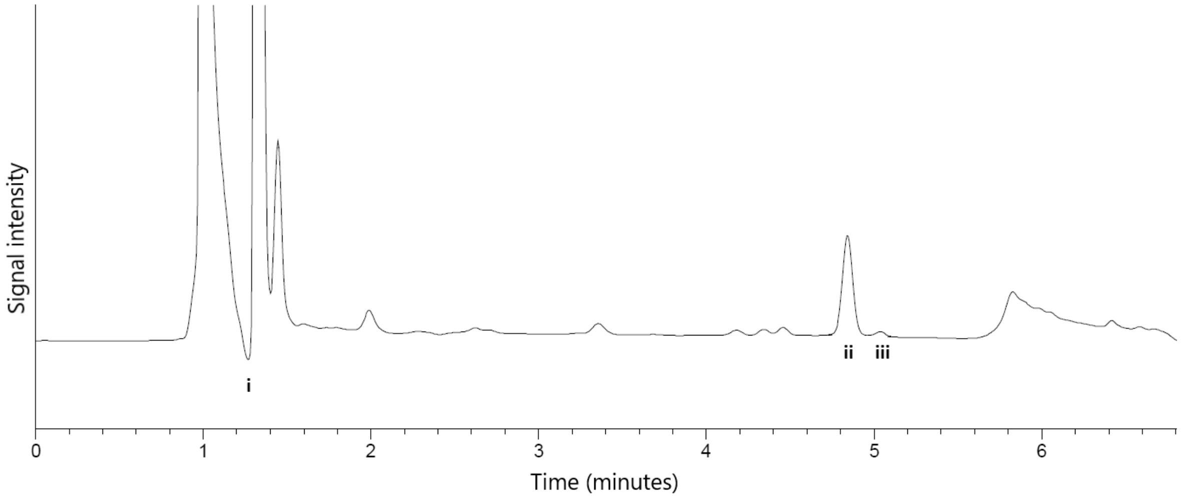

Supplement: Supplementary file 1 [file marinedrugs-20-00634-s001.zip › marinedrugs-1927935-supplementary.pdf]
